# Immunosecurity: immunomodulants enhance immune responses in chickens

**DOI:** 10.5713/ab.20.0851

**Published:** 2021-02-13

**Authors:** Keesun Yu, Inhwan Choi, Cheol-Heui Yun

**Affiliations:** 1 Department of Agricultural Biotechnology and Research Institute of Agriculture and Life Sciences, Seoul National University, Seoul 08826, Korea; 2 Institute of Green-Bio Science and Technology, Seoul National University, Pyeongchang 25354, Korea; 3 Center for Food Bioconvergence, Seoul National University, Seoul 08826, Korea

**Keywords:** Stressor and Immune Response, Chicken Immunology, Antibiotic Growth Promoter, Immunomodulants

## Abstract

The global population has increased with swift urbanization in developing countries, and it is likely to result in a high demand for animal-derived protein-rich foods. Animal farming has been constantly affected by various stressful conditions, which can be categorized into physical, environmental, nutritional, and biological factors. Such conditions could be exacerbated by banning on the use of antibiotics as a growth promoter together with a pandemic situation including, but not limited to, African swine fever, avian influenza, and foot-and-mouth disease. To alleviate these pervasive tension, various immunomodulants have been suggested as alternatives for antibiotics. Various studies have investigated how stressors (i.e., imbalanced nutrition, dysbiosis, and disease) could negatively affect nutritional physiology in chickens. Importantly, the immune system is critical for host protective activity against pathogens, but at the same time excessive immune responses negatively affect its productivity. Yet, comprehensive review articles addressing the impact of such stress factors on the immune system of chickens are scarce. In this review, we categorize these stressors and their effects on the immune system of chickens and attempt to provide immunomodulants which can be a solution to the aforementioned problems facing the chicken industry.

## INTRODUCTION

With the growth of the global population, the consumption of meat and dairy products has also increased. At the same time, animal diseases directly linked with low productivity and potential threats to human health have become a significant public concern. Advances in transportation have facilitated the movement of people, animals, and animal products, which resulted in the easy transmission of these animal diseases. Therefore, endemic animal diseases have become a pandemic trend. For example, avian influenza (AI) was an endemic disease in China until 2004; however, it became a worldwide pandemic due to the transmission of the infection at a trade market and *via* migratory birds. In the United States, more than 50 million poultry animals were killed during the highly pathogenic AI outbreak in 2014 through 2015, which resulted in an economic loss of USD 879 million [1]. In addition, since AI is zoonotic, the pandemic trend of animal diseases can be devastating not only for the productivity of animals but also for human safety. As an attempt to solve the disease problem, antibiotics have been extensively used; however, the use of antibiotics to prevent diseases and promote the growth performance of livestock has been pointed out as one of the main causes for the presence of antibiotic-resistant bacteria that also severely threaten human health. As a matter of fact, the use of antibiotics as a feed additive (antibiotic growth promoter, AGP) has been banned in several countries and is thus decreasing globally. In addition to diseases, various stress factors, such as drastic thermal changes [2], deteriorated environments [3], and nutritional imbalance [4], which also adversely affect domestic animal productivity, are increasingly attracting considerable attention from animal scientists, field managers of animal farms, and operation staff in the livestock industry.

Stressors that negatively affect chicken productivity and their impact on nutritional aspects, including body weight gain (BWG) and feed conversion ratio (FCR), have been reviewed previously [5] and are thus beyond the scope of this review. However, very few studies focus on how the aforementioned stressors relate with the immune system or affect the performance of chicken productivity. The immunology of domestic animals in the modern farming industry is becoming increasingly important, as we could discover answers to the convoluted and urgent questions, including finding suitable antibiotic replacements and immunomodulants that are directly linked to the productivity of domestic animals. Therefore, this review will discuss the effects of nutrition and the gut microbiota on intestinal physiology and the immune system, immunomodulants as alternatives to antibiotics, and, finally, further studies and future directions to solve such problems.

## STRESSORS AND IMMUNE RESPONSE

Numerous studies on feed additives that increase immune activity and disease resistance in chickens have been conducted. As chickens have a relatively shorter breeding period compared with other domestic animals, culling is often employed rather than treatment upon disease outbreak. In addition, the lack of appropriate reagents to conduct studies on chicken immunology indicates that the basic science of the development of immune cells and the roles they play in various organs or against infectious agents in chickens has long been neglected. Thus, the basic research including cells and organs in relation to immune responses coincident with their development at normal condition or inflammation is yet to be defined in detail.

“Stressors” refer to various substances that can induce stress. The immune system, like other physiological reactions in the body, uses proteins and energy to induce inflammation via specific cells and molecules in an adverse relationship with FCR and BWG. Thus, an excessive immune response and inflammation are directly correlated with poor growth performance of chickens. Although stressors vary, the productivity of the animal is closely related to the gut environment and nutrition. This chapter focuses on stressors that affect the gut environment, nutrition, and immune responses in chickens.

### Effects of imbalanced nutrition on the immune system

Nutrients are utilized as a source of energy by living organisms for physiological activity. Therefore, nutritional balance is directly related to animal productivity, including growth performance together with, BWG, FCR, and meat accretion. At the same time, nutrients influence the composition of intestinal microbiota, gut physiology, and mucosal immune responses to the benefit or detriment of chicken productivity. The animal feed constituents can be largely divided into two parts: i) energy sources (carbohydrates, proteins, and fats) and ii) non-energy sources (minerals, vitamins, water, and fibers). As an energy source, corn and soybean meal are commonly used as the main sources of carbohydrates and proteins, respectively [6] .

Non-starch polysaccharides (NSPs), which are found naturally in grains and by-product meals, are a major part of dietary fiber, which includes cellulose, pectins, glucans, gums, mucilages, inulin, and chitin (yet excluding lignin). NSPs exhibit various biological and physiochemical properties, including water dispersibility, viscosity, and bulk effect, interfering with digestibility and converting into short-chain fatty acids (SCFAs) upon fermentation [7] suggested their importance in health implications. Unlike insoluble NSPs, soluble NSP is known to have detrimental effects on intestinal immune responses in chicken. Soluble NSP augments retention time of gut digesta by increasing its viscosity and promoting anaerobic intestinal condition by thickening water layer of mucosa. Those changes induced by inclusion of soluble NSPs in feed favors the potential colonization of undesirable anaerobic microbes in chicken, such as *Clostridium perfringens* (*C. perfringens*) and *Escherichia coli* (*E. coli*), which can cause necrotic enteritis and/or acute inflammation [8]. Furthermore, feed containing high level of soluble NSPs such as rye-based feed has been suggested to cause a high incidence of systemic infection and inflammation due to the increased gut permeability, especially in chicken infected with undesirable bacteria. It was noting that those phenomena can be alleviated by inclusion of *Bacillus*-based direct fed microbial (DFM) in feed since monogastric animals including chicken do not have their own enzymes to break down the β-linkages in soluble NSP while *Bacillus*-based DFM are superior producer of the enzymes such as xylanase that can break down the β-linkages in soluble NSP [9].

Proteins for chicken feed can originate from diverse sources (e.g., soybean meal, cottonseed meal, or fish meal, etc.) with different crude protein levels. It has been suggested that low-protein feed reduces antibody production against sheep red blood cells (SRBCs) [10]. Furthermore, the cell number and proliferation of leukocytes were decreased in chickens fed with protein-restricted feed and infected with *Pasteurella multocida*. These results indicate that dietary protein restriction impacts both humoral and cell-mediated immunity [11]. In addition to the amount of protein, the amino acid composition, which is dependent on the source of the protein, can also affect the immune system by altering the gut microbiota. For example, a high proportion of animal-derived proteins are known to increase the colonization of *C. perfringens* in chicken. When compared with plant-derived protein, animal-derived protein contains a higher amount of glycine and methionine, necessary for the growth of *C. perfringens* and its toxin production, which hampers the digestive enzyme activity [12]. Some amino acids also directly regulate various cellular immune responses, including in intestinal epithelial cells (IECs) and immune cells [13]. Such a regulation is directly driven by the amino acid itself or indirectly by its metabolites. For example, methionine is often sufficient in broiler feed that mainly consists of corn and soybean meal. Chickens fed with insufficient methionine exhibit greater induction of inflammatory cytokines interleukin (IL)-1 and IL-8 in the liver, which is caused by a decrease in lipid transporter and the accumulation of hepatic lipid where methionine serves as an essential signaling molecule [14]. Cysteine, which is a non-essential amino acid, is endogenously synthesized from methionine and is closely related to immune cell proliferation [15].

In chicken feed, fat (normally supplied in liquid form) is the major source of essential fatty acids. It provides the highest caloric value among the energy source nutrients. Although its constituent volume is relatively small in chicken feed, it plays a significant role in not only nutrition but also in immunological aspects, depending on the number of double bonds, the proportion of fatty acids, and the carbon bond lengths in the oil. Its primary functions include the i) enhancement of digestibility by inducing the production of digestive enzymes, such as amylase and lipase [16]; ii) improvement of gut physiology, such as villus height (VH), tight junction formation, and mucin secretion [17]; iii) inhibition of inflammation *via* the downregulation of signaling molecules [18]; and iv) modulation of adaptive immunity *via* increased antigen-specific antibodies following immunization [19]. In addition, oil can minimize dust contamination of the diet and thus minimize safety concerns. The adverse effects of oxidized oil were observed in gut physiology and humoral and cellular immunity [20–22] through the inhibition of tight junction protein production, secretory immunoglobulin (Ig)A, and T cell differentiation. Changes in the immune response induced by oils have been reported, but the precise mechanism of action has seldom been investigated in chicken.

Although vitamins and trace minerals do not have energy value, they are essential for efficient digestion, meat production, and even survival of the animal. Vitamin and trace mineral deficiency often negatively affects the development and maintenance of the host immune system. Vitamin A deficiency results in debilitated cellular immune responses, such as a decrease in CD4^+^ and CD8^+^ intraepithelial lymphocytes (IEL), mitogen-induced splenic lymphocyte proliferation, and reduced serum interferon-gamma (IFN-γ) production during *Eimeria acervulina* infection [23]. Some vitamins and minerals also exhibit similar properties affecting the immune system. Both vitamin E and selenium act as antioxidants, which reduce free radical and lipid peroxidation to control oxidative stress [24]. Supplementary administration of both nutrients maintains the heterophil/lymphocyte ratio in inflammatory conditions and increases the antigen-specific antibody response following immunization [25]. Although the maintenance of the heterophil/lymphocyte ratio in inflammatory conditions is a direct effect of the inhibition of adrenal corticosterone synthesis of vitamin E and selenium [25], whether the increased amount of antibody is a direct effect of these nutrients on B cells or an indirect effect of cytokines produced by T cells in chickens has yet to be addressed. While vitamins and minerals of similar properties often work together, avoidance of direct contact of minerals with vitamins in the feed has been recommended, as mineral oxidization can reduce the efficacy of vitamins [26]. The excess of vitamin A attenuates humoral immunity by impairing the antibody response against Newcastle disease virus (NDV) [27] and also hampers vitamin D absorption [28]. Excessive usage of typical vitamins is likely to cause toxicity and adversely affect the immune system and utilization of other vitamins.

While a number of studies have addressed the issues on diverse nutrients and potential solutions for adverse effects on gut health and the immune system of chickens, it is still unknown how these nutrients precisely affect host physiology. For example, the anti-inflammatory and bactericidal functions of butyrate have been reported in both chicken and mouse models [29,30]. Butyrate has been suggested to induce polarization of macrophages and fatty acid oxidation of T cells in mice; however, in chickens, the precise mechanism for its bactericidal and anti-inflammatory functions has not been reported. In a similar vein, delicate mechanistic studies are required to determine how specific nutrients act on IECs to regulate VH and tight junction proteins, class switching and Ig production, and immune cell differentiation and proliferation in mucosal sites in chickens.

To evaluate the effect of nutrients on immunological aspects, studies often measure the weight of immune organs and total antibodies from serum or intestinal washes. However, these indicators do not fully represent the effect of nutrients on the immune system. This is because the composition and absolute number of immune cells are far more important than the weight of the immune organ as each and every immune cell has a unique function in addition to their general pro- and anti-inflammatory properties. Therefore, it may be of greater value to analyze the changes in the number of specific immune cells within a target organ, along with their functions (i.e., phagocytosis, cytotoxicity, cytokine secretion, and migration). Furthermore, numerous studies have examined total Ig as an indicator of enhanced humoral immunity; however, antigen specificity is essential to T and B cell activities, so antigen-specific Ig should instead be analyzed. Measurements of neutralizing Igs, if possible, would be the best indicator to achieve enhanced humoral immunity.

### Effects of dysbiosis on the immune system

Similar to other animals, the gastrointestinal (GI) tract of chickens is populated with diverse and complex microbiota communities. The interaction and activity of the gut microbiota are closely related with the feed and host cells, as well as the composition of the microbiota communities themselves. The gut microbiota is responsible for various aspects of gut physiology and health, such as the development of intestinal integrity, regulation of adequate immune responses, and competition with harmful or opportunistic pathogens [31]. Furthermore, the gut microbiota facilitates the absorption of nutrients in the GI tract of the host by pre-digesting nutrients. However, in a situation at which the host cannot digest, microbiota may interfere with nutrient absorption by competing with the host and potentially generating toxic substances [32]. Dysbiosis, a condition of imbalanced microbiota, is a devastating stressor that interferes with the absorption of nutrients by host IECs and the development of gut immune system by causing chronic inflammation. Therefore, keeping and providing (if and when feasible) beneficial gut microbiota is especially important in the animal industry to prevent infectious diseases and to improve productivity.

The composition of the microbiota in each GI organ is affected by various factors; accordingly, the immune response is also affected. One of the factors that dramatically influence the chicken gut microbiota is age. A few reports have suggested that the colonization of the gut microbiota in chickens occurs before hatching, although it is fair to say that more studies are required [33]. Unlike mammals, the development of avian embryos occurs outside of the maternal body without any direct interaction. One possible explanation for gut microbiota colonization during the embryonic period is the inclusion of maternal oviduct microbiota with the secretory proteins and the zygote during the formation of the fertilized egg in the oviduct [34]. Early colonization of microbiota after hatching is very important in the health and growth performance of poultry, since perturbation of microbiota with antibiotics in 1-day-old chickens led to decreased small intestinal mucosa production and monocyte/macrophage lineage cells (KUL01^+^) in 2-week-old birds, with a reduced performance of the intestinal immune responses [35]. Similarly, antibiotics treatment at hatching caused a severe reduction in the transcription of IL-10 and IFN-γ and in the number of CD4^+^CD8^−^CD25^+^ and CD4^+^CD8^+^CD25^+^ T cells from the cecal tonsil (CT) in the chicken [36]. As microbiota species diversify, the variation in individuals remains relatively low before 7 weeks of life in commercial chickens [37]. At the phylum level, Bacteroidetes are the major microbial communities in the ceca of 1-week-old chickens, followed by Firmicutes and Proteobacteria. However, Firmicutes have become the major community with the significant reduction of Proteobacteria and Bacteroidetes in 3-week-old chickens [38]. The transcription levels of pro- and anti-inflammatory cytokines by the gut microbiota exhibited both negative and positive correlations in chickens, respectively, in an age-dependent manner. Although the reason and precise mechanism for the alteration of cytokines induced by intestinal microbiota are not fully understood, the trend of studies to date indicates that an immunomodulatory strategy by controlling the gut microbiota at the early time point may be beneficial.

Diet is the source of energy not only for the host but also for the gut microbiota to compete with the host. Therefore, the feed is a factor that significantly influences the changes in the gut microbiota communities in domestic animals. Most nutrients in the feed are usually absorbed by the proximal gut; in the ileum or ceca, where the concentration of microbiota is increasingly high, the nutrients that the host cannot utilize are digested by the microbiota. For instance, NSPs are hydrolyzed by bacteria and primarily fermented into SCFAs, acetate, propionate, and butyrate. These are a source of energy for host IECs and are known to increase the length of jejunum and ileum, VH, and number of goblet cells [39]. Moreover, they tend to enhance anti-inflammatory properties [40] and bactericidal abilities by increasing the transcriptome of the host defense peptides [41] in immune cells. A feed with high levels of NSPs, such as wheat and barley, increases the levels of *Lactobacillus* and coliforms that are likely to produce SCFAs [42]. Conversely, a large amount of low-quality protein in the feed reaches the ceca, which causes an increase in proteolytic bacteria [43]. These bacteria are likely to induce dysbiosis and ferment these proteins, producing toxic substances, such as indole and cresol, thus causing inflammation and intestinal damage [44]. Therefore, control of the gut microbiota through the use of appropriate nutrients and feed additives is important to improve the productivity of domestic animals without causing unnecessary inflammation.

Pathogenic materials could induce the changes in the intestinal microbiota causing dysbiosis, an imbalanced microbiota state. Dysbiosis not only worsens gut physiology such as VH, tight junction formation, and mucin secretion, but also induces an inflammatory response by activating the immune system. Several biomarkers of dysbiosis, such as Proteobacteria and Firmicutes/Bacteroidetes ratio, have been found in humans and mice [45,46], and they could be used similarly in chickens [47]. For example, avian influenza virus (AIV) infection increases the phylum of Proteobacteria, especially *E. coli*, and decreases other probiotic organisms such as *Lactobacillus* and *Enterococcus* [47]. Accordingly, those changes in chickens weakened the gut barrier function and induced an inflammatory response, evidenced by the decrease in transcriptome of tight junction proteins (Zonaoccludin-1, claudin 3, and occludin) and mucin together with increase in pro-inflammatory cytokines (IFN-γ, IFN-α, and IL-22) in IEC, respectively [47]. Although dysbiosis and corresponding changes in the gut immune system have been confirmed in chickens, it is unclear whether the induction of dysbiosis is solely a direct effect due to pathogen infection or an indirect effect by proinflammatory activities, or perhaps the both. In mouse studies, it has been revealed that gut dysbiosis caused by influenza virus infection is induced by IFN-γ secreted by lung-derived C-C motif chemokine receptor (CCR)9^+^ CD4^+^ T cells migrating to the intestine [48]. Therefore, mechanism studies on the relationship among pathogens, dysbiosis, and immune system in chickens should be further investigated.

Factors (age, diet, and pathogens) that induce dysbiosis and negatively affect gut physiology and immune system has been partially identified in chickens. How these factors induce dysbiosis in linked with gut physiology and immune system are yet to be known. For example, heat stress is one of the stressors that greatly affect productivity in the chicken industry. It deteriorates gut physiology, causing leaky gut syndrome by reducing the expression of tight junction proteins [49]. In addition, it reduces the development of lymphoid organs, thereby reducing T and B cells and impairing humoral immunity against antigens [50]. It has been reported that heat stress can induce dysbiosis in chickens. However, the effect of heat stress-induced dysbiosis on the gut physiology and immune system has not been reported. Furthermore, it remains unclear how the population dynamics of the intestinal microbiome change immune system. For example, the exact mechanism of how KUL01^+^ macrophages are reduced when young chickens are treated with antibiotics is largely unknown. It has been demonstrated in mice that intestinal macrophages and dendritic cells (DCs) are maintained by monocyte influx by CCR2 and CCR7. Similarly, chemokine receptor-dependent influx of immune cells can be suppressed in chickens when treated with antibiotics and should be investigated in more detail. More importantly, one must carefully establish strategic approaches when such inhibition of immune cell migration is beneficial to the animal. Collectively, in chickens, comprehensive studies are required to define the precise mechanisms of crosstalk among intestinal microbes, IECs, and immune cells in relation to stressors.

## ANTIBIOTICS AND THEIR SUBSTITUTE

### Ongoing issue with antibiotics

Due to the growth of the global population, meat consumption has increased more than fourfold in the last 50 years. By 2050, the global population is expected to reach 9.7 billion, primarily resulting from the urbanization of developing and under-developed countries. With this urbanization, the demand for meat is going to be definitely increased together with the increased population, however, it will be a formidable task to meet this demand with the current platform and technology [51].

To produce massive amounts of meat products, livestock is often housed and raised in large groups with high densities and therefore often susceptible to infectious agents. Not too long ago, low doses of antibiotics used to be a common feed additive for non-therapeutic purposes for domestic animals, which are known as AGPs [52]. Despite their various advantages, such as growth promotion and potential disease control, the indiscriminate use of sub-therapeutic doses of antibiotics in domestic animals was pointed out as a main cause of the increase in antibiotic-resistant bacteria. For this reason, the use of antibiotics as AGPs has been banned in the European Union, the United States, and some Asian countries (Indonesia, Bhutan, Myanmar, Nepal, Sri Lanka, and Thailand), beginning with South Korea in 2011, only 5 years after the EU movement [53]. China, using antibiotics as AGPs in the largest quantity in the world, is also reducing their use and planning a full ban by the end of 2020 [54].

In the chicken industry, the use of AGPs to improve BWG up to 8% and feed utilization up to 5% has been common since the 1940s [55]. When looking at the known amount of antibiotics used in humans and animals up to date, it is ironic that the mechanism of action of AGPs has never been fully understood. Several direct and indirect lines of evidence have indicated that the beneficial effects of AGPs can be primarily divided into two: effects on bacteria and on the host. Numerous studies have suggested that AGPs have an impact on the profiles of bacterial growth and/or microbial communities. AGPs have been used below the minimum inhibitory concentration (MIC), which is the lowest concentration required to stop the growth of bacteria [52], and therefore different dose of antibiotics, knowing AGP is a sub-MIC, would have different effects on the same bacteria. In fact, it is likely that the effects of sub-MIC antibiotics are not related to the direct inhibition of bacterial growth, although it has been suggested that sub-MIC antibiotics can modify their properties, including the inhibition of the expression of virulence factors often together with biofilm formation and the induction of susceptibility to phagocytosis by host immune cells [56].

It has been reported that chickens treated with AGPs exhibited decreased diversity in their microbial communities in the ceca and ileum [57]. Considering the crucial correlation between microbiota diversity and metabolism, several studies have attempted to elucidate how AGP-induced microbiota alteration impacts their productivity. Bile salt hydrolase (BSH) is an adverse bacteria-produced enzyme that hampers fat digestion and the endocrine system of the host. Low-dose antibiotic treatment reduced *Lactobacillus salivarius* populations and deconjugated bile acid in the ileum, indicating the inhibition of the source of BSH and its products [58]. It has been reported that AGPs can regulate the levels of amino acid, fatty acid, and nucleoside metabolites in chickens, although the exact mechanisms are unknown [59]. These data proposed that AGPs might regulate the gut microbiota communities to be more efficient in nutrient digestion. While the direct relationship between elevated metabolites and BWG has not been fully elucidated in chickens, one possible explanation might be the anti-inflammatory properties of modified metabolites, which are related with muscle mass and BWG by the regulation of catabolic hormones [60].

It has been reported that AGPs also act directly on host cells and exert anti-inflammatory effects on host physiology, including IECs and gut immune cells. Thinner gut walls and increased digestive enzyme activities in chickens fed with AGP-containing feed have been reported [61]. Although the exact mechanism is yet to be determined, one possibility suggested from mice is that innate immunity is suppressed by AGPs and that reduced inflammation can have a beneficial effect on gut wall thickness and enzyme activity [62]. AGPs can increase CD25^+^ IELs, which are known to have regulatory T cell functions in chickens [63] indicating that AGPs may also suppress adaptive immunity.

Collectively, the mechanisms of AGP known thus far are attenuating the virulence of bacteria, benefiting host metabolism, and inducing an anti-inflammatory response in the host. However, to develop an antibiotic substitute with efficiency similar to that of antibiotics, mechanistic studies including the changes in beneficial or harmful microbiota communities together with the effect of metabolites produced by this microbiota linked to the growth performance of chickens should be investigated in detail.

### Immunomodulants as substitutes to antibiotics for growth promotion and disease prevention

*Probiotics*: Probiotics are live microbes known to provide beneficial effects on a host×s health and immunity when administered in an adequate amount. As presented in Table 1, probiotics can benefit hosts in several ways, such as the following: by modulating the composition of the gut microbiota toward a more favorable composition for host well-being, such as increased proportions of host-beneficial lactic acid-producing bacteria *Lactobacillus* and *Bifidobacterium*; preventing pathogen colonization by competitive adherence; and by producing certain metabolites that strengthen the barrier function of the gut epithelium and modulating the immune responses of the host [64].

Traditionally, research on probiotics in chickens was conducted to improve BWG and productivity. Various species and strains of probiotics were administered to chickens as feed additives, which resulted in increased BWG [65]. Increased BWG seems to closely relate to a healthy intestinal barrier, increased VH, and VH/crypt depth (CD) ratio, which provides better chances of nutrient absorption and is an indicative parameter for better gut morphology. Higher VH and VH/CD ratio can be dependent on the proliferation of the gut epithelial cells, which is inhibited by high concentrations of ammonia produced from bacteria in chickens fed with dried *Bacillus subtilis* [66]. It has been reported that the administration of probiotics in chickens reduced urease activity, which converts urea into ammonia in the gut, thus resulting in low ammonia levels and proliferation of the gut epithelial cells [67]. This indicates that treatment with probiotics in chickens promotes epithelial cell proliferation by reducing the ammonia levels coincident with low urease activity, thus resulting in improved BWG and productivity.

It has been reported that chickens administered with probiotics exhibited better immune responses to invading pathogens. The stimulation of chicken macrophages *in vitro* with various *Lactobacillus* species increased the transcription of inflammatory cytokines (IL-1β and tumor necrosis factor-α) and molecules (interferon regulatory factor 7 [IRF7], 2×-5×-oligoadenylate synthetase [OAS], and interferon induced transmembrane protein 3 [IFITM3]) involved in the antiviral responses against AIVs, indicating that probiotics can enhance the antiviral capacity of macrophages against AIV infection [68]. Furthermore, supplementation of probiotics induced high phagocytic ability in increased heterophils in response to *Salmonella* infection [69], which is correlated with low mortality and better protection from this infection.

Since probiotics have attracted considerable attention as a substitute for AGP, numerous studies on the effects of probiotics on humoral immunity, which is closely related to the efficacy of vaccines, have been conducted. Large numbers of studies that used various kinds of probiotics, mainly *Lactobacillus* species and *B. subtilis*, have confirmed an increase in systemic IgG and mucosal IgA titer in chickens immunized with live-attenuated or inactivated forms of pathogens responsible for major poultry diseases, such as NDV, AIV, and *Salmonella* [70,71]. Although the precise mode of action involved in enhanced antibody titer following vaccination has not yet been fully elucidated in chickens, mouse and human studies suggested that probiotics can promote the activation of DCs, resulting in the CD4^+^ T cell-mediated differentiation of IgA-secreting B cells and thus increase in IgA titer [72].

Collectively, the use of probiotics as immunomodulants in chicken could be a desirable method for improving host immunity against pathogens, as probiotics can promote the barrier function of the GI tract and enhance host immunity, including phagocytic ability, antiviral innate immunity, and pathogen-specific antibody responses. Although many studies have reported the effects of probiotics on the immune systems of chickens, it is still largely unknown how certain combinations of probiotic strains protect against infectious diseases directly and/or indirectly *via* host cells. Therefore, further studies on how different probiotic species impact immune responses against certain pathogens should be conducted in chickens. Moreover, it should also be confirmed whether precise mechanisms known to elicit improved immune responses in mouse models are similarly involved in chickens fed with probiotic supplements.

*Polysaccharides*: Polysaccharides are widely used and studied immunomodulants in chickens. Many studies that used prebiotics and β-glucan have been conducted. Prebiotics are feed ingredients, mainly composed of poly- or oligosaccharides, which cannot be broken down by host digestive enzymes but are instead used to promote growth and/or activity of certain intestinal bacteria. As presented in Table 2, the beneficial effects on the host×s health and immunity of prebiotics include the changes in the composition of microbiota, gut morphology, antigen-specific cellular and humoral immune responses, and inflammation state, endowing enhanced defensive ability against invading pathogens [73].

The most noticeable changes reported in chickens treated with prebiotics were the composition of certain gut microbiota, with increased “host-friendly” microbes and reduced growth of harmful microbes. The administration of prebiotics increased the proportion of SCFA-producing bacteria, such as *Lactobacillus*, *Fusobacterium*, and *Bifidobacterium* [73]. Prebiotics also suppress the growth of *E. coli* and *Salmonella* species by attaching to the type 1 fimbriae of these pathogens, thereby interfering with the attachment to the mannose receptor on the host cells [74]. Furthermore, chickens administered with prebiotics exhibited increased BWG, which might be explained by the increases in VH, jejunum length, and sufficient time for nutrient absorption, probably as a result of the increased proportion of SCFA-producing bacteria coincident with gut epithelial cell proliferation in the intestine [75].

Prebiotics have also been reported to modulate population, distribution, and effector functions of chicken immune cells, especially those in gut-associated lymphoid tissues [76]. Prebiotics are involved in the development of the immune system. It prepares the immune system to exert immune responses by regulating the recruitment of T and B cells to the chicken×s secondary lymphoid organs, such as the CT, in the early stages of life. It has also been suggested that the population and proliferation of adaptive immune cells became suppressive when treated with prebiotics. For instance, fructooligosaccharide induced a decrease in the proportion of B cells in the CT, with reduced lymphocyte proliferation in response to mitogen concanavalin A [77]. The immunosuppressive effects of these prebiotics can be explained by the hypo-responsiveness of DCs in the CT, which occurs due to the increase in *Lactobacillus* species in the intestine of chickens fed with a diet containing prebiotics [77]. However, many different types of prebiotics induce the upregulation of both the innate and adaptive immune responses. Various kinds of prebiotics promote the expression of pro-inflammatory cytokines, such as IL-1β and IL-17A, in the gut at a steady state [75,78]. These phenomena indicate that prebiotic supplementation in chickens could help to enhance the protective functions through IL-17A in the gut, which plays a significant role in maintaining intestinal epithelial integrity. It is important to supplement an adequate dose of prebiotics to ensure proper protective immune response in chicken since the excessive dose of prebiotics in feed can act negatively on growth performance [79]. In this regard, 3.37% of galactooligosaccharide (GOS), in starter stage feed and 1.685% of GOS in grower and finisher feed in chickens are reported as adequate amounts without causing harmful effect [78]. Based on the light of the fact that prebiotics enhanced growth performance in chicken while ensures the induction of inflammatory mediators, IL-1β and IL-17A [78], use of appropriate amount of prebiotics could induce positive effects on overall performance. It is probable that prebiotics could induce an increase in pro-inflammatory cytokines from innate immune cells, including γδ T cells. Prebiotics also improve and prolong humoral immune responses against intruding infectious agents, such as AIV, NDV, and infectious bursal disease virus (IBDV), with a significantly high level of antigen-specific antibodies [80]. Although there are numerous studies that describe the modulating effect of prebiotics on the gut microbiota and immune responses of chickens, most studies are focused on changes in genus levels such as *Lactobacillus* and *Bifidobacterium*. It should be noted that various species exist in the same genus, and thus, it is necessary to study the relationship between the species affected by prebiotics and, at the same time, the host immune cell factors in microbial species level.

β-Glucan is a polysaccharide derived from the cell wall of barley, oat, yeast, fungi, and algae [81]. The supplementation of β-glucan in feed could inhibit the colonization of pathogens in the GI tract as well as modulate immune responses in chickens. β-Glucan is known to inhibit the colonization of *Salmonella enterica* in the chicken gut. It has also been suggested that the reduction in the colonization of pathogens in chickens treated with β-glucan might be caused by increased pathogen clearance by the increased CD8^+^ T cell population in IELs [82]. On the other hand, it could also be that β-glucan favors the adhesion of probiotic bacteria to IECs to compete against pathogen colonization [83]. However, the adhesion of probiotic species to IECs by β-glucan has not yet been reported in chickens that is waiting for the investigation on its, and neither has any other precise mechanism of action.

Several studies also demonstrated that β-glucan enhanced the phagocytic and antimicrobial activities of phagocytes, such as macrophages and heterophils in chickens [81]. The mode of action for the enhanced phagocytic and bactericidal activities coincident with the increase in nitrite and IL-1β in chicken phagocytes treated with β-glucan can be explained indirectly by human and mouse studies. It has been demonstrated that specific receptors, such as complement receptor 3 or dectin-1, are expressed on human and mouse phagocytes, resulting in increased bactericidal abilities of phagocytes [84]. However, studies on whether chicken macrophages and heterophils also express these β-glucan receptors have not yet been conducted; related downstream signaling cascades must still be elucidated. The administration of β-glucan in chickens also induced an increase in SRBC-specific antibody responses coincident with the enhanced CD4^+^ helper T cells [82].

In addition to the aforementioned immunomodulatory functions, β-glucan has been known to induce trained immunity. Trained immunity can be defined as altered secondary responsiveness of the innate immune system against mostly external stimuli, including lipopolysaccharide (LPS), chitin, and bacterial or fungal cells themselves, which provoked by metabolic reprogramming and epigenetic modifications of innate immune cells. The epigenetic changes in trained immunity include chromatin modification and altered levels of histone acetylation and methylation, which are the two major modifications that function as specific transcription regulators, which result in subsequent long-term changes in immune cell metabolism. This phenomenon was discovered and is typically studied in monocytes, which exhibit altered level of inflammatory responses and expression levels of molecular markers of monocytes.

In chickens, the evidence suggested an induction of trained immunity in monocytes treated with β-glucan using an *ex vivo* experimental model. Chicken primary monocytes treated with β-glucan exhibited increased production of nitric oxide compared with cells treated with LPS. Simultaneously, the expression of major histocompatibility complex class II and CD40, indicating immunological activation, was also upregulated [85]. These results indicate that β-glucan can drive the immune system to better deal with invading foreign substances, although further mechanistic studies using *in vivo* models are necessary to validate metabolic and epigenetic modulations.

In conclusion, polysaccharides induce positive effects on chickens in terms of both growth performance and immune responses. These positive effects are likely caused by the ability of polysaccharides to increase the proportion of beneficial SCFA-producing bacteria and to reduce harmful bacteria in the host. Previous studies revealed that the administration of polysaccharides to chickens can enhance immune responses and inhibit excessive inflammation, although the mechanisms remain unknown. Therefore, more comprehensive studies are required to elucidate in detail the mechanisms, explaining how changes in the gut microbiota in chickens induce alterations in immunological responses, such as trained immunity, resulting in the protection against pathogens.

*Egg yolk IgY antibodies*: Antigen-specific IgY isolated from egg yolk has been suggested as an antibiotic substitute for the prevention of targeted pathogens. IgY is similar to the mammalian IgG antibody in terms of its function and occupies. It comprises most of the antibodies existing in chicken serum and egg yolk. The oral administration of antibodies has been reported in various livestock, including chickens [86]. Most of the mechanisms of action of the protective abilities of IgY against various bacteria (*E. coli*, *C. perfringens*, *Campylobacter jejuni*, and *Salmonella*) [87] and *Eimeria*, a parasitic protozoa that causes coccidiosis, have been identified through the use of *in vitro* models [88]. The major action of orally administered IgY is to neutralize target pathogens. Specifically, IgY attaches to antigens and interferes with the adhesion of bacteria to the IECs. It also impairs bacterial cellular signaling, thereby attenuating the growth and toxin production [89]. However, the precise mode of action mechanism has not been confirmed in chickens.

Orally administered IgY could have advantages over antibiotics as it targets the intended pathogens only, whereas antibiotics may kill multiple microbiota, including commensal and beneficial bacteria. Moreover, with IgY treatment, there would be no fear of generating antibiotic-resistant bacteria. However, due to the low resistance to proteolytic enzymes, such as pepsin and denaturation at low pH, problems with the normal function of IgY in the small intestine have been pointed out. To solve this problem, IgY has been encapsulated in microcapsules to increase the delivery to the small intestine and bactericidal efficiency [90].

Collectively, IgY has the advantage of being able to specifically eliminate pathogens harmful to the host. Moreover, it could likely be used as a substitute for antibiotics for disease prevention. However, to be practically and more efficiently used in chickens, mechanistic and functional studies are absolutely required, and a solution to the delivery problem must be further defined.

*Antimicrobial peptides*: Antimicrobial peptides (AMPs), also called host defense peptides, are a part of the innate immune responses found among most, if not all, classes of life, including mammals, birds, amphibians, plants, fungi, and bacteria [91]. AMPs are short chains of about 6 to 50 amino acids linked by peptide bonds, which usually have a positive charge and amphipathic feature (i.e., containing both polar and nonpolar portions in its structure).

AMPs have anti-bacterial properties against highly virulent pathogens, such as *Salmonella* and *Campylobacter*, as well as immune boosting effects on chickens. The mode of action of AMPs in chickens is relatively well known compared with the other immunomodulants. For instance, positively charged AMPs are easily attracted to negatively charged bacterial cell components and thereby exert their bactericidal functions [91]. Once AMPs are attached to the bacterial cell surface, they can form pores on the surface of the target bacterium, thus causing lysis by perturbing the osmotic balance of the intra- and extra-bacterial environment. AMPs can also penetrate the bacterial cells to disturb the synthesis of nucleic acids or proteins and the cell cycle of the bacteria, thus inhibiting their proliferation [91]. On the other hand, AMPs also appear to have a direct interaction with immune cells. A chicken AMP (AvBD8) induced the expression of pro-inflammatory cytokines (IL-1β and IL-12p40) and chemokines (C-C motif chemokine ligand [CCL] 4, C-X-C motif chemokine ligand 13 [CXCL13], and CCL20) in chicken macrophages *via* the activation of the mitogen-activated protein kinase signaling pathway [92]. Apart from their effects on chicken macrophages, AMPs are also known to induce increases in the IEL population in the duodenum, jejunum, and ileum as well as enhance antibody production after IBDV vaccination [93].

It has been reported that the expression of AMP can be regulated by interactions with other kinds of immunomodulants like probiotics and prebiotics. Certain probiotics strain, such as *Lactobacillus rhamnosus* MLGA, induced an increase of AMP (AvBD-9) in chicken’s IECs through toll-like receptor-2 dependent manner [94]. Chickens fed with a feed containing β-glucan also upregulated expression of various AMPs in both of mucosal and systemic perspectives including AvBD-10 in jejunum, and cathelicidin (cath)-1, AvBD-2, -4, and -7 in spleen [95]. These phenomena suggest that certain immunomodulants can control expression of AMPs in chicken, and thus, regulate immunomodulating effect of AMPs.

Collectively, AMPs have the potential to prevent disease in chickens by either directly killing pathogens or boosting the immune cell number and activation of the expression of inflammatory cytokines, chemokines, and antibodies. The mechanism of action of AMPs in the direct killing of bacteria and enhancing the activity of immune cells is relatively well defined in *in vitro* models. However, these must be further elucidated in chickens, which leave room for *in vivo* studies to establish the potential of AMPs as efficient immunomodulants and alternative for AGPs. Further investigation for the interaction between AMP and other immuomodulants might be needed for better understanding of comprehensive immunomodulating mechanisms of AMPs and aforementioned immunomodulants in chicken.

## CONCLUSION

In conclusion, numerous studies have suggested that nutrients are not only energy sources for organs and tissues but also components of substances related to gut physiology and immunity, such as in the production of tight junction proteins, mucins, chemokines, and cytokines. Moreover, they determine the characteristics of immune cells *via* the regulation of metabolism. Metabolites are produced by microbiota and the host and, in some cases, interfere with the absorption of nutrients by the IECs. Nutrients can also regulate gut immunity by directly interacting with the IEC and immune cells. Imbalanced nutrition and microbiota can break the intestinal homeostasis of the host, thus causing inflammation, and also utilize the energy in unnecessary biosynthesis. Interactions among nutrients, microbiota, and gut immunity are not limited to local sites and eventually affect systemic homeostasis that is responsible for the entire body, surely the growth performance.

**Figure 1:**
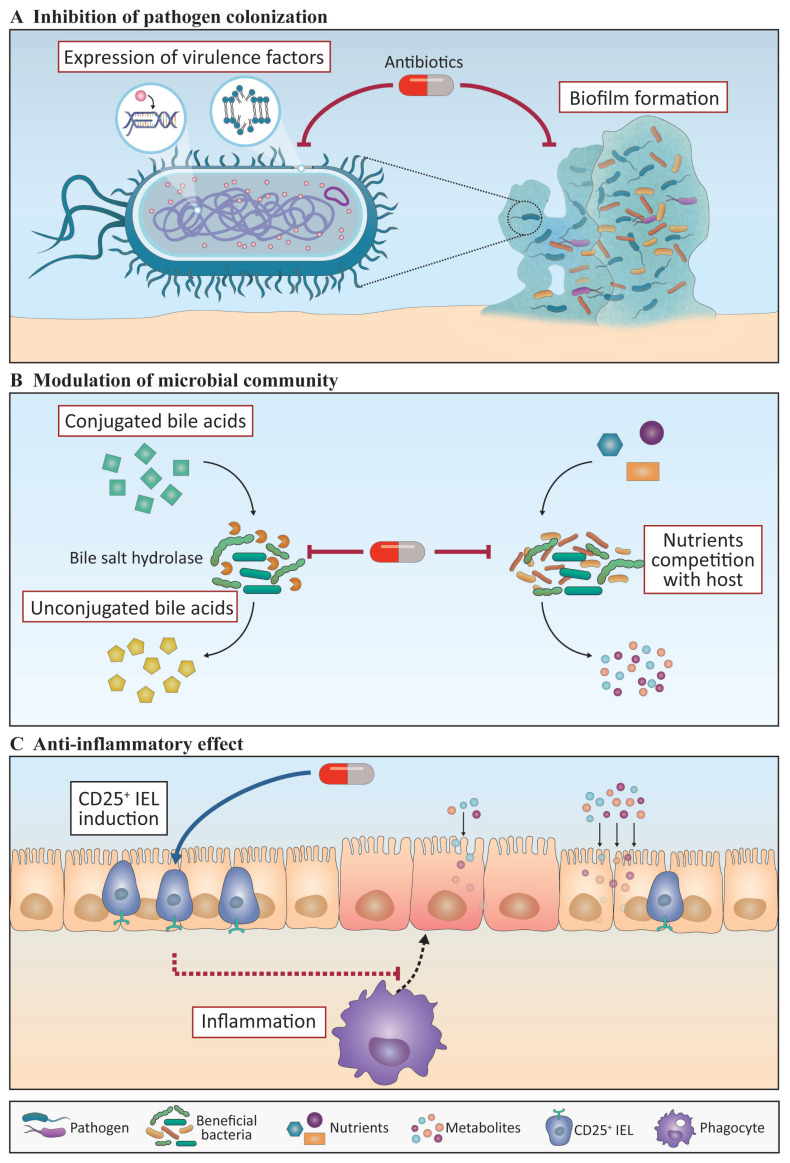
Graphical summary of the mechanism of action of antibiotics. Antibiotics could modulate the gut microbiota and host immune responses. (A) Antibiotics control pathogen colonization through the reduction of virulence genes and proteins associated with their movement, attachment, and biofilm formation. (B) Antibiotics modulate advantageous microbial communities through the reduction of bacteria that produce enzymes that interfere with host digestion, such as bile salt hydrolase (BSH). They also inhibit the competition of nutrients between the gut microbiota and the host. (C) Antibiotics induce an anti-inflammatory response by increasing the number of CD25^+^ intraepithelial lymphocytes (IEL), which could reduce the inflammatory responses that contribute to the reduced thickness of the gut wall. Broken lines indicate the mechanisms elucidated in mouse studies that have not yet been identified in chicken. The contents in the black and red boxes represent phenomena that are induced or suppressed by substances, respectively.

**Figure 2:**
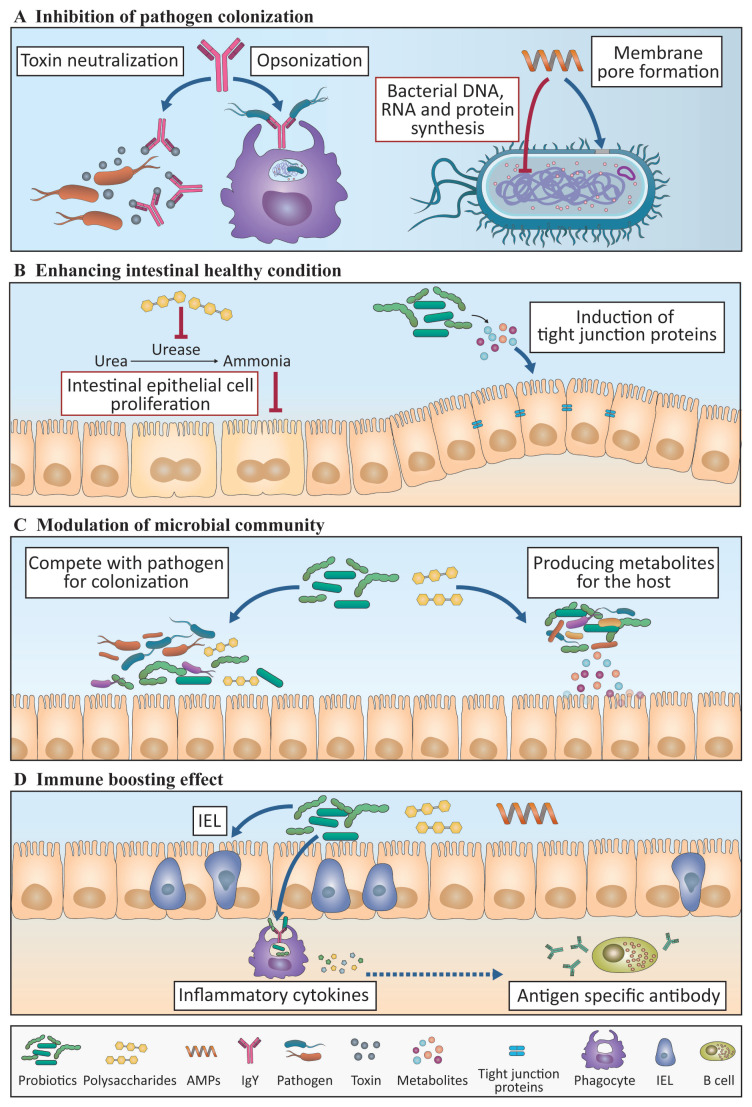
A schematic diagram showing the mode of action of immunomodulants. Immunomodulants that have beneficial effects on the gut microbiota and immune response are shown. (A) Antimicrobial peptides (AMPs) control pathogens by disturbing the bacterial membrane and inhibiting bacterial DNA, RNA, and protein synthesis. IgY inhibits toxin neutralization and opsonization. (B and C) Probiotics and polysaccharides induce an increase in beneficial bacteria, such as *Lactobacillus* and *Bifidobacteria*, which compete with pathogens for adhesion. They also induce the production of beneficial bacterial metabolites that function in the intestinal epithelial cells (IECs) to increase the villus height:crypt depth (VH:CD) ratio and tight junction proteins and thus improve gut health. (D) AMPs, probiotics, and polysaccharides also have immune boosting effects by increasing phagocytosis and pro-inflammatory cytokine production in innate immune cells, increasing the number of intraepithelial lymphocytes (IELs), and increasing the production of antigen-specific antibodies from the different class of B cells. Broken lines indicate the mechanisms elucidated in mouse studies that have not yet been identified in chicken. The contents in the black and red boxes represent phenomena that are induced or suppressed by substances, respectively.

We have shown how antibiotics (Figure 1) and immunomodulants (Figure 2) affect host growth performance and the immune system. Probiotics and polysaccharides have the ability to regulate the gut microbiota community and host immune responses, thus affecting the growth performance, whereas IgY and AMPs may have the potential to prevent diseases. Therefore, numerous studies are being conducted using these products as substitutes for AGPs and are expected to yield beneficial effects in productivity, with the enhancement of BWG and trained immunity. However, most studies in chicken are currently observation-based, and there is no sufficient evidence to explain the modes of action. Thus, the precise mechanisms by which AGPs and immunomodulants act on the gut microbiota, as well as the systemic and mucosal immune responses in the host, should be investigated in greater detail. It should be noted that the absence of basic studies on the nutritional immune responses in chickens, for instance, the developmental process of various immune cells and their roles at a steady state or in inflammatory conditions in conjunction with nutritional status, must be rectified in the future.

## FUTURE PERSPECTIVE

It is true that most, if not all, producers at animal farms try very hard to avoid inflammation reactivity, as many have experienced adverse effects on BWG, low feed intake, and utilization of unnecessary energy in domestic animals. However, one should keep in mind that avoiding and suppressing inflammation not always play a beneficial role in enhancing the growth performance of domestic animals. For instance, it is well known that suppression of the inflammatory response during vaccination results in decreased antibody titers [96], indicating the importance of inflammation to the enhancement and maximization of vaccine efficacy. Therefore, the use of anti-inflammatory reagents in animals around the time of vaccination is not recommended.

The vaccination schedule for broilers usually ends 4 weeks after hatching, whereas that for layers lasts about 18 weeks. Considering that the efficacy of the NDV vaccine increases when inflammatory substances, such as LPS or poly I:C, are injected together [97], immunomodulants can be beneficial, particularly around the time of vaccination in broilers. In case of layers, a series of vaccines against the NDV, infectious bronchitis, Marek disease, infectious laryngotracheitis, infectious bursal disease, avian encephalomyelitis, and fowl pox are administered up to 18 weeks after hatching. Considering the relatively long-term vaccination schedule of layers with such diverse types of vaccines, the application of immunomodulants may be a viable option in terms of cost-effectiveness and minimization of unexpected side effects, yet well suited for the purpose. Most of all, sanitation is of paramount importance, as the inflammatory responses induced by harmful pathogens would hamper all aforementioned benefits of immunomodulants.

To meet the growing need for animal products and to fight the pandemic trend of animal diseases, both fundamental and basic studies on pathogenesis and immunological mechanism as well as field studies in animal farms should be conducted as a proof of concept. Furthermore, the regulation and guidelines set by authorities for satisfying the needs of producers and consumers for domestic animals are equally important. Government support for the development of safe and effective antibiotic alternatives that prevent pandemic diseases outbreaks must be considered as one of the top priorities. The last, but not the least, these efforts of academia, the animal industry, and the government authorities should be in harmony to resolve these issues.

## Figures and Tables

**Table 1:** Effect of probiotics on gut physiology and immune system in chicken

Genus	Species (strain)	Effects	References
*Lactobacillus*	Mixture of *L. salivarius*, *L. johnsonii*, *L. reuteri*, *L. crispatus*, *L. gasseri*)	Increased α-proteobacteria and decreased Firmicutes in ceca Increased serum IgG level against H9N2	[98]
	*L. acidophilus* (JTBo8) or *L. salivarius* (JTBo9)	Increased IL-1β and interferon regulatory factor 7 in macrophages	[68]
	*L. salivarius* (L61, L55)	High phagocytic index in heterophils	[69]
	*L. acidophilus* (D2/CSL)	Increased antibody titer against NDV	[99]
	Antigen expressing (EtMIC2, HA) recombinant *L. plantarum*	Increased CD3^+^CD4^+^ and CD8^+^ T cells	[100]
*Bacillus*	*Bacillus coagulans*	Increased *Lactobacillus* and *Bifidobacterium* while decreased *Salmonella* in ceca Increased number of goblet cells	[101]
Other probiotics	*Paenibacillus polymyxa* (BSC10)	Increased levels of *MUC2*, *CLDN1*, *OCLN* genes	[68]
	*E. faecium* (NCIMB 10415)	Increased mucosal IgA against *Salmonella Enteritidis* vaccination	[70]

IgG, immunoglobulin G; H9N2, influenza A virus subtype haemagglutinin (HA)9 and neuraminidase (NA)2; IL-1β, interleukin-1β; NDV, Newcastle disease virus; EtMIC2, *E. tenella* microneme-2; *MUC2*, mucin 2; *CLDN1*, claudin-1; *OCLN*, occludin.

**Table 2:** Effect of polysaccharide on gut physiology and immune system in chicken

Type of prebiotics	Effects	References
Galactooligosaccharide (GOS)	Increased *Lactobacillus* species in gut	[102]
	Increased expression of *MUC6* and *CLDN1* genes in jejunum and cecum	[75]
	Upregulation of IL-17A and IL-1β in jejunum and cecum	[75,78]
Fructooligosaccharide (FOS)	Decreased *Helicobacter* and *Desulfovibrio* in ileal mucosa	[103]
	Increased *Lactobacillus* in gut	[102]
	Decreased B cell proportion in cecal tonsil Decreased mitogen responsiveness of lymphocyte	[77]
Mannanoligosaccharide (MOS)	Increased *Butyricimonas* and *Roseburia* in cecum	[104]
	Down-regulation of IFN-γ in cecal tonsil in *Salmonella* infection	[74]
Xylooligosaccharide (XOS)	Increased *Bifidobacterium* in cecal contents Increased villus height and crypt depth ratio and longer length of jejunum	[105]
Inulin	Increased heterophils:lymphocytes ratio	[106]
	Colonization of CD8α^+^ T cells and γδ^+^ T cells in cecal tonsil	[76]
	Inhibition of IgG retardation against sheep red blood cells	[107]
Yeast cell wall extract β-Glucan	Decreased proportion of *E. coli* and *Salmonella* in cecum	[108]
	Inhibited colonization of *Salmonella enterica* in chicken gut Enhanced proliferation and phagocytic activity of macrophages and heterophils	[81]

*MUC6*, mucin 6; *CLDN1*, claudin-1; IL, interleukin; IFN-γ, interferon-gamma; IgG, immunoglobulin G.
